# Characteristics of cardiac toxicity after definitive radiation therapy for thoracic esophageal cancer in Japanese patients

**DOI:** 10.1093/jrr/rraf056

**Published:** 2025-09-23

**Authors:** Keita Tsukahara, Takanori Abe, Satoshi Saito, Takumi Sakaguchi, Jun Watanabe, Misaki Iino, Tomomi Aoshika, Yasuhiro Ryuno, Genta Michimata, Tomohiro Ohta, Mitsunobu Igari, Ryuta Hirai, Yu Kumazaki, Shin-ei Noda, Shingo Kato, Yutaka Miyawaki, Hiroshi Sato

**Affiliations:** Department of Radiation Oncology, International Medical Center, Saitama Medical University, 1397-1, Yamane, Hidaka, Saitama 350-1298, Japan; Department of Radiation Oncology, International Medical Center, Saitama Medical University, 1397-1, Yamane, Hidaka, Saitama 350-1298, Japan; Department of Radiation Oncology, International Medical Center, Saitama Medical University, 1397-1, Yamane, Hidaka, Saitama 350-1298, Japan; Department of Radiation Oncology, International Medical Center, Saitama Medical University, 1397-1, Yamane, Hidaka, Saitama 350-1298, Japan; Department of Radiation Oncology, International Medical Center, Saitama Medical University, 1397-1, Yamane, Hidaka, Saitama 350-1298, Japan; Department of Radiation Oncology, International Medical Center, Saitama Medical University, 1397-1, Yamane, Hidaka, Saitama 350-1298, Japan; Department of Radiation Oncology, International Medical Center, Saitama Medical University, 1397-1, Yamane, Hidaka, Saitama 350-1298, Japan; Department of Radiation Oncology, International Medical Center, Saitama Medical University, 1397-1, Yamane, Hidaka, Saitama 350-1298, Japan; Department of Radiation Oncology, International Medical Center, Saitama Medical University, 1397-1, Yamane, Hidaka, Saitama 350-1298, Japan; Department of Radiation Oncology, International Medical Center, Saitama Medical University, 1397-1, Yamane, Hidaka, Saitama 350-1298, Japan; Department of Radiation Oncology, International Medical Center, Saitama Medical University, 1397-1, Yamane, Hidaka, Saitama 350-1298, Japan; Department of Radiation Oncology, International Medical Center, Saitama Medical University, 1397-1, Yamane, Hidaka, Saitama 350-1298, Japan; Department of Radiation Oncology, International Medical Center, Saitama Medical University, 1397-1, Yamane, Hidaka, Saitama 350-1298, Japan; Department of Radiation Oncology, International Medical Center, Saitama Medical University, 1397-1, Yamane, Hidaka, Saitama 350-1298, Japan; Department of Radiation Oncology, International Medical Center, Saitama Medical University, 1397-1, Yamane, Hidaka, Saitama 350-1298, Japan; Department of Gastroenterological Surgery, International Medical Center, Saitama Medical University, 1397-1, Yamane, Hidaka, Saitama 350-1298, Japan; Department of Gastroenterological Surgery, International Medical Center, Saitama Medical University, 1397-1, Yamane, Hidaka, Saitama 350-1298, Japan

**Keywords:** radiotherapy, esophageal cancer, cardiac toxicity

## Abstract

In recent years, there has been growing interest in cardiac toxicity following radiation therapy (RT) for esophageal cancer; however, detailed incidence and risk factors in Japanese patients remain unclear. The purpose of this study was to clarify the incidence, timing, risk factors, and dose-volume relationships of multiple cardiac toxicities, including pericardial effusion, heart failure, arrhythmia, cardiac valve disease and acute coronary syndrome. We retrospectively analyzed patients of thoracic esophageal cancer without distant metastasis who were treated with curative RT at our hospital between 2007 and 2020. Cardiac toxicity events were graded according to common terminology criteria for adverse events v5.0. Association between cardiac dose-volume parameters and grade 2 or higher toxicity was analyzed using logistic regression analysis. The analysis included 250 patients, with a median follow-up period of 21 months. The 2-year cumulative incidence of grade 2 or higher pericardial effusion, heart failure, arrhythmia, and acute coronary syndrome were 36.6%, 0.4%, 1.4%, and 1.3%, respectively. Logistic regression analysis identified the volume of the whole heart receiving 30Gy as a significant risk factor for grade 2 pericardial effusion (OR, 1.03; 95% confidence interval [CI], 1.01–1.04; *P* < 0.01) and grade 2 arrhythmia (OR, 1.10; 95%CI, 1.02–1.18; *P* = 0.01). We reported detailed profile of cardiac toxicity in Japanese patients who received curative RT for esophageal cancer. Reducing cardiac radiation dose may reduce the risk of pericardial effusion and arrhythmia.

## INTRODUCTION

Multimodal approaches including radiation therapy (RT) have improved treatment outcomes for esophageal cancer [1]. For early stage esophageal cancer, RT serves as an organ-preserving treatment option for patients that decline surgery [2]. In cases of locally advanced or inoperable tumors, RT remains the only definitive treatment option [3, 4]. As treatment outcomes and survival times improve across all tumor stages, it is increasingly important to reduce treatment-related toxicity. Cardiac toxicity, including pericardial effusion, heart failure, arrhythmia, and acute coronary syndrome (ACS), is one of the major late complications of RT for esophageal cancer and can significantly impact patient prognosis and quality of life [5, 6]. As a result, the field of cardio-oncology, which seeks to identify and mitigate risk factors for cardiac toxicity during cancer treatment, has gained attention [7]. Previously reported risk factors for cardiac toxicity after RT for esophageal cancer include cardiac dose-volume parameters and various patient factors such as body size, sex, and comorbidities [5]. Previous studies in Japanese patients treated with RT for esophageal cancer report grade 2 or higher pericardial effusion in 19–52% of cases and grade 3 or higher in 4–10% [8–14]. Similarly, the incidence of grade 3 or higher ACS was reported 1–9% [8–10]. However, these studies focused on specific types of cardiac toxicity or included relatively small patient cohorts, and no comprehensive study has evaluated all cardiac toxicities in a large Japanese patient population. In this study, we conducted the largest single-institution analysis of consecutive esophageal cancer patients treated with RT, evaluating the incidence, timing, risk factors, and dose-volume relationships of cardiac toxicities, including pericardial effusion, heart failure, arrhythmia, cardiac valve disease and ACS.

## MATERIALS AND METHODS

### Patients

Patients with pathologically confirmed thoracic esophageal cancer without distant metastasis who underwent definitive RT between 2007 and 2022 were retrospectively analyzed. The follow-up period was defined as from the start of definitive RT to the last confirmed date of survival. Patients with a history of radiotherapy to the chest or with a follow-up period of <6 months were excluded. All patients underwent esophagogastroduodenoscopy (EGD) and chest-abdominal computed tomography (CT) to tumor stage using the Tumor-Node-Metastasis (TNM) classification system of malignant tumors 8th edition. Treatment recommendations, including endoscopic resection, surgery and RT, were carefully determined through a multidisciplinary tumor board comprising thoracic surgeons, thoracic medical oncologists, radiologists and radiation oncologists. This study was approved by our Institutional Review Board (Reference number: 2023–1494) and performed in accordance with the Declaration of Helsinki. Written informed consent for the use of medical data was obtained from most patients; otherwise, an opt-out method was applied, wherein details of the research were published on our website, providing patients an opportunity to decline participation.

### Radiation therapy

For treatment planning, CT images were acquired in both the expiration and inspiration phases. The gross tumor volume (GTV) was delineated on both sets of images and merged to create the internal GTV (iGTV). For cases of superficial tumor not visualized on CT, a metallic clip was placed on the superior and inferior border of the tumor by a thoracic oncologist prior to CT acquisition to aid in GTV delineation. The clinical target volume (CTV) was generated by adding a margin of 2 cm in superior–inferior direction and 0.5 cm in the anterior–posterior and right–left directions to the iGTV to account for microscopic tumor extension. When no evidence of tumor invasion was observed, anatomical structures such as the bones, trachea, heart and great vessels were excluded from the CTV. A planning target volume margin of 5 mm was added to CTV to compensate for setup error. Patients from the 2000s commonly used elective nodal irradiation, but following reports [15] about irradiation field size optimization, involved field RT became the standard from 2010 onward. The most widely used irradiation technology was three-dimensional conformal RT, but intensity modulated RT was used for patients treated after 2020. For superficial esophageal cancer (cT1b), a dose of 60 Gy in 30 fractions was used based on the JCOG0502 regimen. For resectable locally advanced cases (cT2–3), 60 Gy in 30 fractions was initially used (JCOG9906 regimen), while more recently, 50.4 Gy in 28 fractions has been adopted following the JCOG0909 regimen. In unresectable locally advanced cases (cT4), 60 Gy in 30 fractions based on the JCOG0303 regimen was selected as the standard treatment. However, there were some deviations from these protocols, such as dose reduction in cases where adverse events were a concern, or dose escalation in cases where concurrent chemotherapy was not feasible, in order to improve local control. Dose constraints were as follows: lung volume receiving ˃20 Gy (lung V_20_) <35%, spinal cord maximum dose <50 Gy, esophagus volume receiving ˃40 Gy. Initially, no cardiac dose constraints were applied, but in recent years, efforts have been made to limit mean heart dose to <15–20 Gy when possible. The cases treated with three-dimensional conformal radiation therapy (3D-CRT) using a four-beam arrangement comprising anterior–posterior and two oblique fields.

### Chemotherapy

Concurrent chemotherapy included cisplatin/5-fluorouracil and FOLFOX (folinic acid, fluorouracil, and oxaliplatin). Some patients who were initially planned for surgery but subsequently underwent an unplanned transition to chemoradiotherapy, neoadjuvant chemotherapy (docetaxel/cisplatin/fluorouracil or carboplatin/etoposide) was administered.

### Follow up

Patients underwent follow-up CT scan and EGD within 4–6 weeks of completion of RT, with tumor response assessed according to RECIST criteria. If a complete response is achieved, patients achieving a complete response (CR) were considered for boost chemotherapy or followed without adjuvant therapy. Patients without a CR were considered for salvage endoscopic therapy or salvage surgery.

### Evaluation of cardiac toxicity

Cardiovascular events, including such as pericardial effusion, heart failure, arrhythmia, cardiac valve disease and ACS, were considered as cardiac toxicity and were graded according to CTCAE v5.0. Individual cardiac structures (whole heart [WH], left ventricle, right ventricle, left atrium, right atrium, left anterior descending artery, left circumflex artery, right coronary artery) were contoured on the radiation treatment plan using the University of Michigan Contouring Atlas [16]. Dose-volume parameters (mean dose, V5–50 Gy) were assessed for each cardiac structure.

### Statistical analysis

Cox proportional hazards model was used to analyze the association between patient characteristics and the occurrence of late cardiac toxicity. The association between cardiac dose-volume parameters and grade 2 or higher cardiac toxicity was analyzed using logistic regression analysis. In the analysis of the cumulative incidence of cardiac toxicity, Gray’s test was used considering the possibility that death could be a competing risk. A *P*-value of <0.05 was considered statistically significant. All statistical analyses were conducted using IBM SPSS Statistics for Windows, version 25.0 (IBM Corp., Armonk, NY, USA).

**Table 1:** Patient and tumor characteristics (N = 250)

Characteristics	Number of patients	%
Sex		
Male	215	86.0
Female	35	14.0
Age		
<70	105	42.0
≥70	145	58.0
ECOG PS		
0	239	95.6
1	11	4.4
Diabetes		
Yes	33	13.2
No	227	90.8
Pre-existing cardiac disease		
yes	41	16.4
No	209	83.6
Pathology		
SCC	243	97.2
ADC	6	2.4
NEC	1	0.4
Tumor location		
Upper	32	12.8
Middle	164	65.6
Lower	54	21.6
Clinical Stage (UICC8th)		
I	38	15.2
II/III	88	35.2
IV	124	49.6
Concurrent chemotherapy		
Yes	169	67.6
No	81	32.4
Radiation dose		
<50.4 Gy	145	58.0
≥50.4 Gy	105	42.0
Modality		
3D-CRT	248	99.2
IMRT	2	0.8

Abbreviations: ECOG PS, Eastern Cooperative Oncology Group performance status; SCC, Squamous cell carcinoma; ADC, Adenocarcinoma; NEC, Neuroendocrine carcinoma; UICC, Union for International Cancer Control; 3D-CRT, three-dimensional conformal radiation therapy; IMRT, Intensity-modulated radiation therapy.

## RESULTS

**Fig. 1:**
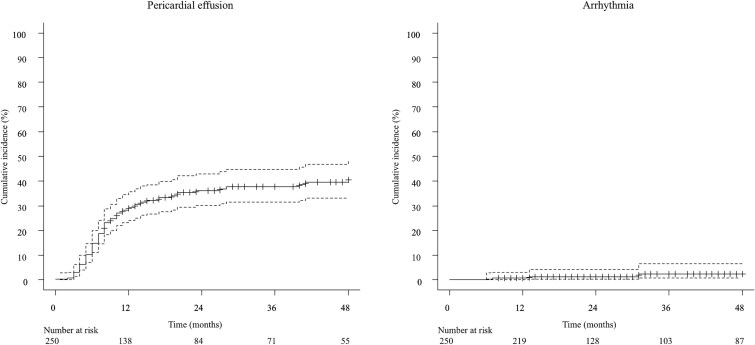
The cumulative incidence of grade 2 or higher pericardial effusion and arrhythmia are shown. The 2-year cumulative incidence of pericardial effusion and arrhythmia was 36.6% and 1.4%, respectively.

**Fig. 2:**
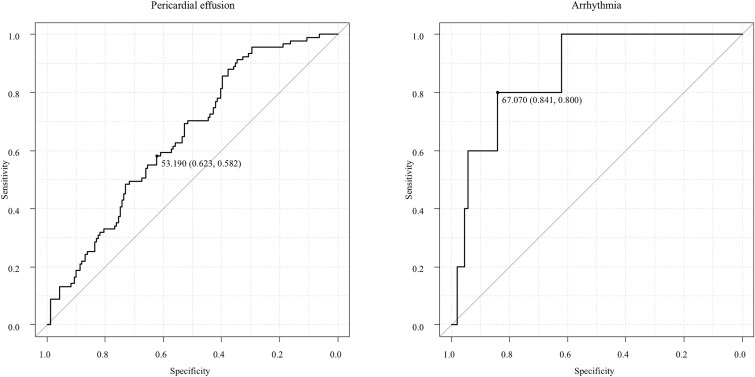
Left panel: ROC curve for WH-V30Gy and pericardial effusion grade 2 or higher (AUC = 0.65; 95% CI, 0.58–0.72; *P* < 0.01). The cut off value was 53%. Right panel: ROC curve for WH-V30Gy and arrhythmia grade 2 or higher (AUC = 0.87; 95%CI, 0.74–0.98; *P* < 0.01). The cut off value was 67%.

**Fig. 3:**
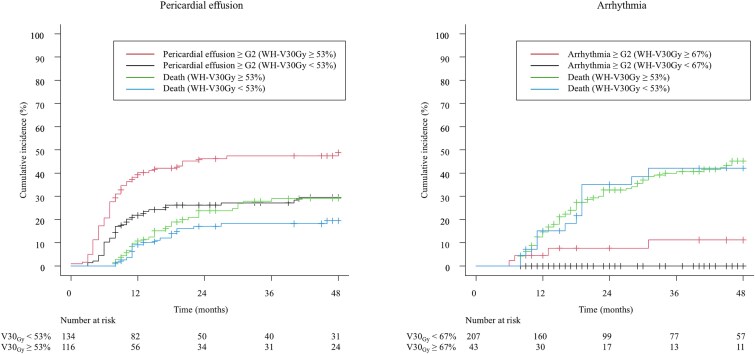
Left panel: cumulative incidence of pericardial effusion grade 2 or higher and death divided into two groups based on WH-V30Gy (<53% and ≥53%) using the Gray’s Test. The red line shows the cumulative incidence of WH-V30Gy ≥53% group, with a 2-year cumulative incidence of 47%. The black line shows the WH-V30Gy <53% group, with a 2-year cumulative incidence of 27%. As a result of the Gray’s test, a statistically significant difference was observed in the cumulative incidence of pericardial effusion between the two groups (*P* < 0.01), whereas no significant difference was found in the cumulative mortality (*P* = 0.13). Right panel: cumulative incidence of arrhythmia grade 2 or higher and death divided into two groups based on WH-V30Gy (<67% and ≥67%) using the Gray’s Test. The red line shows the cumulative incidence of WH-V30Gy ≥67% group, with a 2-year cumulative incidence of 7.6%. The black line shows the WH-V30Gy <67% group, with a 2-year cumulative incidence of 0.0%. As a result of the Gray’s test, a statistically significant difference was observed in the cumulative incidence of arrhythmia between the two groups (*P* < 0.01), whereas no significant difference was found in the cumulative mortality (*P* = 0.85).

Patient characteristics are shown in Table 1. A total of 250 patients were retrospectively analyzed with a median follow-up period of 21 months. Squamous cell carcinoma accounted for 97% of patients, and the most common tumor location was the middle thoracic esophagus (65.6%). Nearly half of patients presented with stage IV disease (49.6%). Five patients (2.0%) were originally scheduled for surgery and therefore underwent neoadjuvant chemotherapy including Docetaxel. A total of 169 patients (67.6%) underwent concurrent chemotherapy, and the median radiation dose was 50.4 Gy. Details of cardiac toxicity are shown in Table 2. Grade 2 or higher cardiac toxicity was observed in 98 patients (39%), with a median time to onset of 13 months. Notably, only 11 of these patients (11% of the 98 cases) experienced events beyond 24 months. No patient characteristics were significantly associated with grade 2 or higher cardiac toxicity, which was shown in Table 3. Pericardial effusion was the most common event, with 91 patients (36.4%); the 2-year cumulative incidence was 36.6%, shown in Fig. 1. The whole heart dose parameters were significantly associated with grade 2 or higher pericardial effusion, shown in Table 4. In the ROC analysis, WH-V30Gy has the largest AUC (AUC = 0.65; 95% CI, 0.58–0.72; *P* < 0.01, cut-off values of 53%) which was shown in Fig. 2. The Gray’s test results for the cumulative incidence of the two groups separated by cut-off value of WH-V30Gy, Fig. 3.

**Table 2:** Grade 2 or higher cardiac toxicity

	Grade 2	Grade 3	Grade 4	Grade 5
Pericardial effusion, n (%)	89 (35.6)	2 (0.8)	0 (0)	0 (0)
Heart failure, n (%)	0 (0)	1 (0.4)	1 (0.4)	0 (0)
Arrhythmia, n (%)	3 (1.2)	2 (0.8)	0 (0)	0 (0)
Cardiac valve disease, n (%)	1 (0.4)	1 (0.4)	1 (0.4)	0 (0)
Acute coronary syndrome, n (%)	0 (0)	2 (0.8)	1 (0.4)	0 (0)
Total, n (%)	92 (36.8)	7 (2.8)	2 (0.8)	0 (0)

**Table 3:** Cox regression analysis for grade 2 or higher cardiac toxicity

Variable	HR	95%CI	*P*-value
Sex: Male vs Female	1.35	0.72–2.53	0.35
Age: <70 vs ≥70	1.31	0.87–1.98	0.2
ECOG PS: 0 vs 1	1.24	0.45–3.38	0.68
Diabetes: Yes vs No	0.73	0.38–1.40	0.35
Pre-existing cardiac disease: Yes vs No	0.97	0.57–1.66	0.91
Tumor location: Upper vs Middle/Lower	1.56	0.79–3.11	0.2
Concurrent chemotherapy: Yes vs No	0.89	0.59–1.36	0.6
Radiation dose: <50.4Gy vs ≥50.4Gy	1.01	0.73–1.64	0.65

Abbreviations: HR, hazard ratio; 95%CI, 95% confidence interval; ECOG PS, Eastern Cooperative Oncology Group performance status.

**Table 4:** Logistic regression analysis for Grade 2 or higher pericardial effusion and arrhythmia

	Pericardial effusion					
	Univariate			Multivariate		
Variable	OR	95%CI	*P*-value	OR	95%CI	*P*-value
MHD	1.05	1.02–1.08	<0.01	1.05	1.03–1.08	0.01
WH-V5Gy	1.02	1.01–1.03	<0.01	1.02	1.01–1.04	0.02
WH-V10Gy	1.02	1.01–1.03	<0.01	1.02	1.01–1.04	0.01
WH-V20Gy	1.02	1.01–1.04	<0.01	1.02	1.01–1.04	0.01
WH-V30Gy	1.03	1.01–1.04	<0.01	1.03	1.01–1.04	<0.01
WH-V40Gy	1.02	1.01–1.04	<0.01	1.03	1.01–1.04	0.04
WH-V50Gy	1.03	1.01–1.05	<0.01	1.04	1.02–1.06	0.06
	**Arrhythmia**					
	**Univariate**			**Multivariate**		
**Variable**	**OR**	**95%CI**	***P*-value**	**OR**	**95%CI**	***P*-value**
MHD	1.17	1.03–1.32	0.02	1.17	1.03–1.32	0.04
WH-V5Gy	1.10	1.00–1.21	0.06			
WH-V10Gy	1.10	1.01–1.20	0.03	1.10	1.01–1.20	0.04
WH-V20Gy	1.09	1.01–1.18	0.03	1.09	1.01–1.18	0.04
WH-V30Gy	1.10	1.02–1.18	0.01	1.10	1.02–1.19	0.02
WH-V40Gy	1.08	1.02–1.15	0.01	1.08	1.01–1.15	0.03
WH-V50Gy	1.07	1.02–1.13	<0.01	1.07	1.02–1.13	0.08

Abbreviations: MHD, Mean Heart Dose; WH, Whole Heart; V5/10/20/30/40/50Gy, Percentage of volume irradiated with 5/10/20/30/40/50Gy.

Arrhythmias were uncommon and included paroxysmal supraventricular tachycardia in 3 patients (1.2%) and atrial fibrillation in two patients (0.8%); the 2-year cumulative incidence was 1.4%, shown in Fig. 1. The whole heart dose parameters were significantly associated with grade 2 or higher arrhythmia, shown in Table 4. In ROC analysis, WH-V30Gy was significantly associated with grade 2 or higher arrhythmia (AUC = 0.87; 95% CI, 0.74–0.98; *P* < 0.01, cut-off values of 67%, respectively), shown in Fig. 2. Gray’s test results for the cumulative incidence of the two groups separated by cut-off value of WH-V30Gy, shown in Fig. 3. In contrast, no significant correlation was noted between left atrial dose parameters and arrhythmia occurrence, shown in Supplementary Table 1.

Regarding ACS, no significant correlation was noted between dose parameters and the occurrence of events, shown in Supplementary Table 2. The same was true for other events, including heart failure and cardiac valve disease.

Considering the shorter follow-up duration in stage 4B cases without distant metastases (median: 19 months), an additional subgroup analysis was conducted including only the 133 stage I-IVA cases (median follow-up: 28 months), shown in Supplementary Fig. 1.

## DISCUSSION

In our study, the 2-year cumulative incidence of grade 2 or higher cardiac toxicity was 38%, with pericardial effusion being the most common event (36.6%). These findings align with previous reports in Japanese patients that found an incidence of grade 2 or higher pericardial effusion ranging from 35% to 52% [13, 14]. The 2-year cumulative incidence of grade 2 or higher heart failure, arrhythmia, and ACS were 0.4%, 1.4%, and 1.3%, respectively. Compared with the results of studies from the United States, the number of ACS and arrhythmia events after RT was lower in our cohort [17, 18]. This may be attributed to differences in baseline cardiovascular risk factors between Japanese and Western populations [19]. However, with Westernization of dietary habits and an aging population, the incidence of heart disease in Japanese patients might increase, making radiation-induced heart disease an emerging concern.

In our study, cardiac dose-volume parameters such as WH-V30Gy were associated with grade 2 pericardial effusion. The ROC analysis determined cut-off values ​​of WH-V30Gy of 53% for pericardial effusion. These findings are consistent with previous studies showing an association between WH-V30Gy and pericardial effusion [20]. Because pericardial effusion occurs as a result of pericardial inflammation, it might be reasonable that whole heart dose is associated with events. Regarding arrhythmia, WH-V30Gy of 67% was significantly associated with arrhythmic events. We also evaluated dose of sub-structure of heart but there was no association with events. However, several reports suggest a relationship between atrial fibrillation and left atrial dose [17, 21, 22]. Therefore, we think that it is still important to reduce the dose to the whole heart as well as the substructure of the heart to reduce arrhythmia. In this study, there was no association between ACS dose-volume parameters. However prior studies have suggested an association between ACS and coronary artery dose and left ventricular dose [9, 23]. The absence of a correlation between ACS and cardiac dose-volume parameters may be due to the limited number of events.

The current study has some limitations. As a retrospective, single-institutional study, selection bias is possible. The median follow-up period of 21 months is relatively short and may lead to an underestimation of cardiac toxicities. However, previous studies have suggested that radiation-induced cardiac toxicity in patients with esophageal cancer tends to occur relatively early, with most events developing within 2 years after treatment [18, 24, 25]. Therefore, it is likely that most clinically significant events were captured within the observation period of the present study. To address concerns related to limited follow-up in advanced-stage disease, a subgroup analysis was performed excluding patients with stage IVB disease, who generally had shorter follow-up durations, shown in Supplementary Fig. 1. Similar results were observed in this subgroup, supporting the robustness of the findings. Nevertheless, it remains difficult to completely eliminate the influence of differences in disease stage or patient background characteristics, which should be acknowledged as a limitation of this analysis.

The potential confounding effect of concurrent chemotherapy could not be fully evaluated in this study. Agents such as 5-fluorouracil and docetaxel have been implicated in cardiac toxicity, and variables including the timing of administration, dosage, number of cycles, and specific chemotherapy regimens may act as confounding factors that influence the observed outcomes. Additionally, cardiac toxicity was not assessed at fixed intervals, which may lead to an underestimation of events. However, because our hospital is an advanced emergency and critical care center, patients presenting with symptoms such as palpitations, shortness of breath, or chest discomfort were promptly evaluated by cardiologists, minimizing the risk of missing a clinically significant cardiac event. In this study, no association was found between patient-related factors and the occurrence of cardiac toxicity, though prior research has identified pre-existing heart disease as a risk factor for cardiac toxicity [18]. Patients with a history of cardiac disease may require closer monitoring following RT.

We clarified the incidence, timing, risk factors, and dose-volume relationships of multiple cardiac toxicities in Japanese patients with esophageal cancer treated with RT. WH-V30Gy was identified as a risk factor for the development of pericardial effusion and arrhythmia. Notably, the incidence of arrhythmia and ACS differed significantly from reports from Western countries, underscoring the importance of population-specific risk analysis.

## Supplementary Material

Supplementary_Figure_1_rraf056

Supplementary_table_1_rraf056

Supplementary_table_2_rraf056

## Presentation at a conference

An overview of this study has been presented at a conference [26], and this paper adds new analytical results and detailed discussion.
