# Supplementary figures and images for: Characteristics of cardiac toxicity after definitive radiation therapy for thoracic esophageal cancer in Japanese patients
Source: J Radiat Res. 2025 Sep 23;66(6):645–51. doi: 10.1093/jrr/rraf056 (PMC12648062; doi:10.1093/jrr/rraf056)

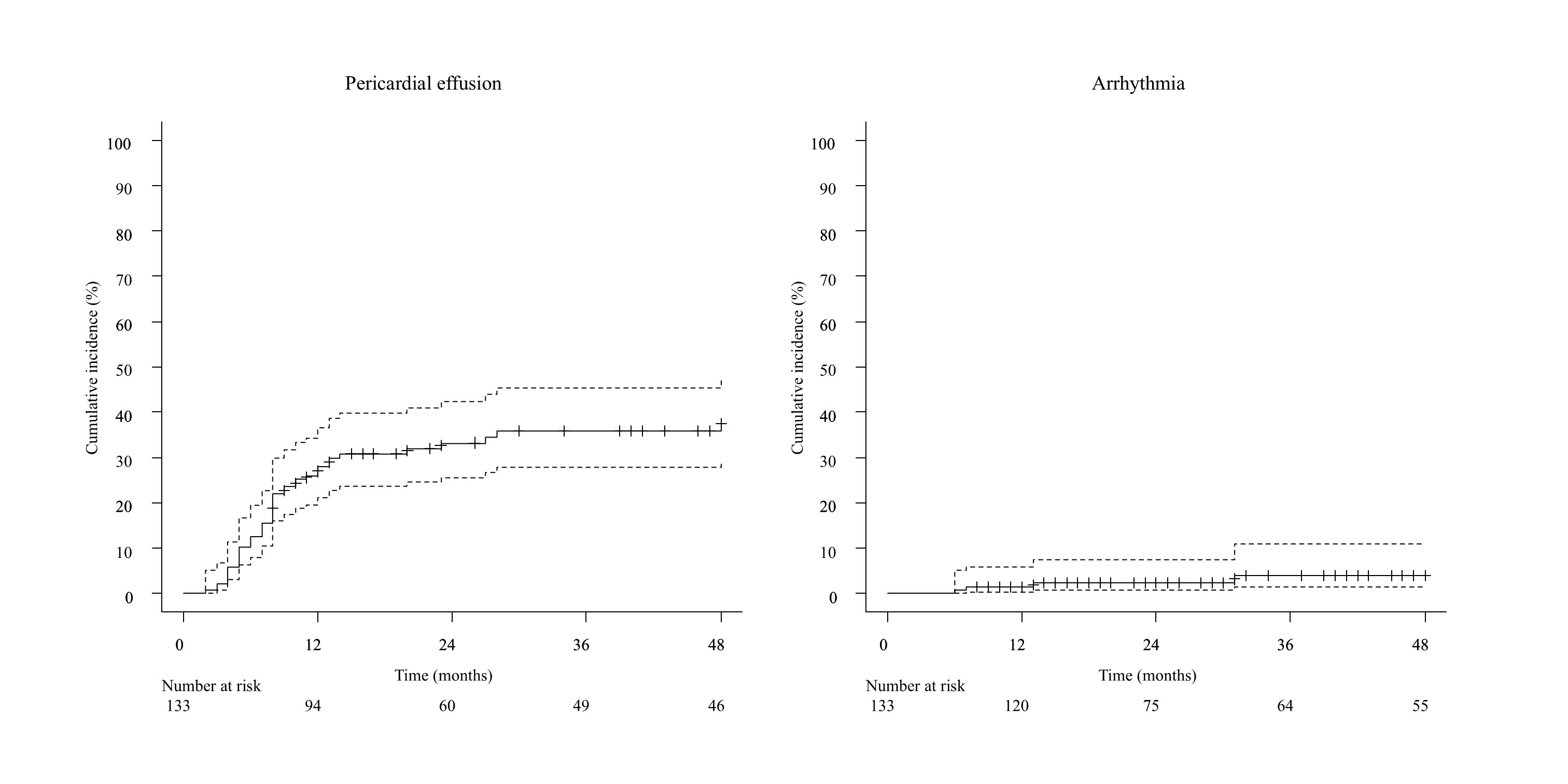

Supplement: Supplementary_Figure_1_rraf056 [file supplementary_figure_1_rraf056.jpeg]
