# Supplementary material for: Characteristics of cardiac toxicity after definitive radiation therapy for thoracic esophageal cancer in Japanese patients
Source: J Radiat Res. 2025 Sep 23;66(6):645–51. doi: 10.1093/jrr/rraf056 (PMC12648062; doi:10.1093/jrr/rraf056)
Supplement: Supplementary_table_1_rraf056 [file supplementary_table_1_rraf056.docx]

Supplementary Table 1.

Logistic regression analysis for Grade 2 or higher arrhythmia and left atrium dose.

|  |  | **univariate** |  |
| --- | --- | --- | --- |
| **Variable** | **OR** | **95%CI** | **p-value** |
| LA-MD | 1.07 | 1.16-2.86 | 0.09 |
| LA-V5Gy | 1.10 | 1.20-2.80 | 0.10 |
| LA-V10Gy | 8.50 | 4011.7-0.46 | 0.50 |
| LA-V20Gy | 1.41 | 2.74-1.05 | 0.30 |
| LA-V30Gy | 1.22 | 1.63-1.74 | 0.19 |
| LA-V40Gy | 1.11 | 1.26-2.35 | 0.13 |
| LA-V50Gy | 1.03 | 1.07-3.63 | 0.06 |

Abbreviations: LA, Left Atrium; MD, Mean Dose
