# Supplementary material for: Characteristics of cardiac toxicity after definitive radiation therapy for thoracic esophageal cancer in Japanese patients
Source: J Radiat Res. 2025 Sep 23;66(6):645–51. doi: 10.1093/jrr/rraf056 (PMC12648062; doi:10.1093/jrr/rraf056)
Supplement: Supplementary_table_2_rraf056 [file supplementary_table_2_rraf056.docx]

Supplementary Table 2.

Logistic regression analysis for Grade 2 or higher ACS and coronary artery dose.

|  |  | **univariate** |  |
| --- | --- | --- | --- |
| **Variable** | **OR** | **95%CI** | **p-value** |
| LAD-MD | 1.04 | 0.96-1.13 | 0.31 |
| LAD-V5Gy | 1.03 | 0.98-1.09 | 0.23 |
| LAD-V10Gy | 1.02 | 0.98-1.07 | 0.25 |
| LAD-V20Gy | 1.02 | 0.99-1.06 | 0.25 |
| LAD-V30Gy | 1.02 | 0.99-1.06 | 0.20 |
| LAD-V40Gy | 1.02 | 0.98-1.05 | 0.34 |
| LAD-V50Gy | 1.02 | 0.98-1.05 | 0.40 |

Abbreviations: LAD, Left Anterior Descending artery.
